# Knowledge about Diabetes and Glycemic Control among Diabetic Patients in Saudi Arabia

**DOI:** 10.1155/2020/1239735

**Published:** 2020-03-07

**Authors:** Noura A. Abouammoh, Muteb A. Alshamrani

**Affiliations:** ^1^Family and Community Medicine, King Saud University, Riyadh 4545/145111, Saudi Arabia; ^2^Family and Community Medicine, Security Forces Hospital, Riyadh 3643/11481, Saudi Arabia

## Abstract

The prevalence of diabetes in various regions has attracted significant attention of the medical experts. The prevalence of diabetes is expected to increase in the future due to changes in lifestyle and unhealthy diets of individuals. The objective of the study is to identify the extent of knowledge related to diabetes and glycemic controls in various diabetic patients living in Saudi Arabia. A total of 435 patients were recruited using a random sampling technique, while following a cross-sectional design. Patients' knowledge was tested using the Michigan Diabetes Knowledge Test. Findings of the study illustrated that the problem was common among middle-aged male patients. A significant amount of knowledge related to the consumption of medicines, insulin, healthy diet, etc. was found among diabetic patients. Despite the fact that people have adequate knowledge, valuable attention is yet required to provide necessary counselling to people living in Saudi Arabia that may help them to control health risks and mortality.

## 1. Introduction

In the healthcare domain, the most proliferated disease recognized across the world is diabetes. This is evident from the increased reporting of diabetes disease which is expected to reach a figure of 366 million [1] and expected to become the seventh leading cause of death by 2030 [2]. In 2014 alone, its global prevalence was reported to be 8.5 percent [2]. The most common type of diabetes is type 2 which is observed among 90 to 95% of the diabetic population globally [3]. It has stimulated as a global health concern accounting to the highest rate of morbidity and mortality [4]. Fareed et al. [5] demonstrate that the deficiency of the insulin actions in type 2 diabetes adds to the macrovascular and microvascular complication, which mitigates the health-related quality of life (HRQoL).

The prevalence of diabetes in developing countries is relatively high. In fact, Saudi Arabia as a developing country has the highest diabetes prevalence in both regional and global landscapes [6]. The state invested about 1,142 million dollars for overcoming the progressive disease in the country which is tremendously affecting its workforce. Sabbah and Al-Shehri [7] highlighted the report of WHO stating that the country spends about $800 for providing care of its country diabetes mellitus person, whose yearly cost reaches up to $9.6 billion. Earlier researches have advocated that the increased prevalence of the disease has not been mitigated despite the massive investment made by the country. Fatema et al. [8] and Mohammadi [9] pinpointed that despite the advancement and development of the disease research, the patient's level of disease knowledge remains low affecting the glycemic control. It has been claimed by Marathe et al. [10] that escalated diabetes complication risk and mortality can be lowered by intensive glycemic control. Alsulaiman et al. [11] indicated that about two-thirds of Saudi diabetic patients have poor glycemic control. On the contrary, glycemic control is an essential indicator to prevent the chronic metabolic disease complications such as metabolic syndrome, cardiovascular and kidney disease, and diabetes. Among chronic metabolic diseases, glycemic control management has been a domain of interest from the past years. The glycemic index shows an increasing fasting blood glucose level after consuming a high carbohydrate-containing diet. The glycemic index is directly associated with different chronic metabolic diseases among the human populations [12].

Similarly, to gain a better understanding of the patient's health literacy and practices, a Malay Elderly Diabetes Self-Care Questionnaire (MEDSCaQ) was also developed by Ishak et al. [13]. The findings of the survey using this questionnaire revealed that health literacy and disease management improved with the presence of family support as well as good diabetes knowledge. The ultimate outcomes of the study include diabetes knowledge, ethnicity, family support, and comorbidities that contributed to the improvement of the disease knowledge. Likewise, paperwork by Shareef [14] stated that disease outcomes improved with pharmacist-administrated diabetes knowledge. It showed that adequate access to diabetes knowledge adds to diabetes management and a patient's control. Diabetes is a condition anticipated for rapid advancement over time. The high blood sugar can influence various cells and organs in the body if type 2 diabetes goes untreated. Side effects include eye damage, stroke, heart disease, and kidney damage. Family members disseminate genes that make them prone to possess type 2 diabetes and to be overweight [15].

Pérez-Escamilla et al. [16] examined the effect on type 2 diabetes patients' glycemic control by introducing an intervention led by the community health worker. In this regard, they used the Diabetes among Latinos Best Practices Trial (DIALBEST) and showed that lifestyle trend and ethnicity contribute to the effective prevention of the disease and improvement of glycemic control.

In a similar context, many researches have attempted to examine the factors which can contribute to the improved health of the patients stimulated by their adequate knowledge of the disease and ineffective glycemic control. The American Diabetes Association in 2015 introduced a diabetes self-management education and support (DSMES) program, which studied patients' attributes and characteristics for providing them with adequate support and customized care. It includes knowledge about their convictions, ethnic practices, sociodemographic factors, and socioeconomic factors [17]. The same study also underlined that supplementing adequate knowledge of diabetes requires the provision of better support for achieving better health outcomes. Zainudin et al. [18] revealed that a gap in the disease knowledge leads to the adaptation of adverse activities affecting the patients' health outcome. Azreena et al. [19] further argued and stated that adequate health literacy is a prerequisite for effective utilization of the measures for efficient diabetes management. Sanal et al. [20] highlighted that glycemic control is substantially associated with diet and physical activity.

The increased disparity in the knowledge concerning diabetes and glycemic control contributes to the ineffective clinical control which impacts the better disease knowledge. Since optimal disease knowledge is integral for reducing the related risk factors of inadequate control, the assessment of the patient's mindfulness is critical. Therefore, the study is aimed at assessing the overall diabetes knowledge in the patients of Security Forces Hospital in Saudi Arabia. The study used a 14-item Michigan Diabetes Knowledge Test (MDKT) questionnaire for testing the knowledge about diabetes among the patients in Security Forces Hospital, Riyadh.

## 2. Material and Methods

### 2.1. Participants and Statistical Sample Size Formula

The study uses a cross-sectional study design for assessing the relationship between type 2 diabetic patients' knowledge and glycemic control in Security Forces Hospital, Riyadh, Saudi Arabia. The period for the study was from January 2018 to March 2018. A total of 435 patients who visited the outpatient clinics of Security Forces Hospital, Riyadh, were recruited using a random sampling method. Random sampling in research methods denotes a variety of selection techniques in which sample participants are chosen by chance, but with an identified likelihood of selection.

### 2.2. Inclusion and Exclusion Criteria

The selected patients were evaluated for the inclusion criteria which involved them being diagnosed for type 2 diabetes, under care at the hospital, and above 18 years. Patients suffering from physical or mental impairment and pregnant women were not included in the study. The sample size was calculated based on the patients admitted in the outpatient clinics of Security Forces Hospital, 95% confidence interval, and 80% power size using G-power version 3.1.9.4.

### 2.3. Tools and Validity and Reliability

The structured questionnaire of the 14-item MDKT was used for examining the consenting participant's diabetic knowledge. HbA1c was collected from the patients' hospital record. The knowledge of diabetes and HbA1c levels were taken as dependent variables. The independent variables involve gender, age, qualification, marital status, time since diagnosis, occupation, and present medication regime. Demographic details of patients were collected through a designed questionnaire that consists of questions related to gender, age, marital status, type of job, educational level, duration of diabetes, etc. Other information includes type of treatment provided to diabetes patients and level of education related to diabetes along with their family history of diabetes.

### 2.4. Questionnaire Filling Method

A self-administered 14-item MDKT questionnaire was used for gathering patients' diabetic knowledge and their basic demographic details. This instrument was used to evaluate the knowledge of the elderly in this study. The Michigan Diabetes Research and Training Center has developed the MDKT. The 14-item questionnaire is reliable and valid and thus has been majorly utilized for evaluating diabetes knowledge. The instrument is comprised of three sections. The first section collected sociodemographic data, the second section consists of the 14-item MDKT, and the third section consists of the laboratory data to be collected from the patients' records. This questionnaire originally held 23 items, though the study only included the first 14 items which address the general awareness of the diabetic disease. These items were inclusive with one right answer along with multiple wrongs. The Arabic translation of the questionnaire was obtained from the study of Azreena et al. [19] for sustaining context relevance, after the approval of his team. The patients selected the questionnaire language.

These items were assessed for their reliability which was found to be 0.7 and 0.71 along with adequate internal consistency (Cronbach alpha), i.e., 0.702. Developed by the University of Michigan's Diabetes Research and Training, the MDKT was used, which is also accessible online (http://diabetesresearch.med.umich.edu/Tools_SurveyInstruments.php#dkt). The study was commenced after achieving authorization from the Diabetes Care Center at Riyadh Security Forces Hospital. The patients were also communicated with the study details and were included after the attainment of their written consent.

### 2.5. Statistical Analysis

The collected data were analyzed using IBM SPSS version 25. Categorical variables were analyzed descriptively. Frequencies and percentages were used to present continuous and categorical variables. The Kruskal-Wallis test was used to show the association between HbA1c levels and sociodemographic characteristics. A haemoglobin A1c (HbA1c) test measures the amount of glucose related to haemoglobin. It is the part of red blood cells that transmits oxygen from the lungs to the rest of the body. An HbA1c test indicates what the average amount of blood sugar connected to haemoglobin has crossed the time period of 3 months. It is a 3-month average because that is usually a red blood cell life. Different chronic conditions can be caused such as kidney disease, nerve damage, and heart disease if HbA1c levels are high.

## 3. Results

Table 1 shows the demographic details of the patients. Among 435 participants, the majority of the participants were male, i.e., 53.1%, whereas the percentage of females was 46.9%. The majority of the samples belong to Saudi nationality (98.6%) and the least of the samples to non-Saudi nationality (1.3%). Much of the sample population belongs to the age group 51-60 years (39.5%), followed by 41-50 years (22.1%) and 61-70 years (16.4%), producing a median age of 55 years. Most of the participants were married (93.3%) as compared to single (6.7%). The education level of the patients showed that the majority held only primary-level education (29.2%) followed by secondary-level education (27.1%) and college degree (26.9%). Family history revealed that 71.9% had a family history of glycemic control, whereas 28.1% did not. The occupation analysis revealed that 38.9% had retired, 33.5% are nonworking, and 23.9% are employed in the government sector while 23.9% worked in the private sector.

Table 2 shows the findings of the examined patient's record. The analysis showed that the majority of the patients were on medication, where 44.8% take oral medicine and 13.8% take insulin therapy alone, while 36.4% took a combination of oral and insulin therapy, whereas dietary interventions were used by 5%. The diabetes family history was found for 72% of the patients, and a diagnosis age median was 11 years, where the majority reported living with diabetes for 6-15 years. Among 435 patients, the majority of the patients (71.2%) have used diabetes knowledge for disease management. The high HbA1c levels were found among the patients (9.3 ± 4.6%). A total of 53.3% reported poor glycemic control of levels above 8.5%, while moderate glycemic control was shown by 1.8%, and lastly, glycemic control below 7.0% level was observed in 14.0% patients.

The percentage of the participants who had correctly answered the questions in the MKDT questionnaire has been depicted in Table 3. It demonstrates that the difference in the normal and special diet is recognized by only 40%, whereas 51.5% understood the significance of the diabetic diet in the health context. A large portion of the sample showed that they considered diabetic diet to be rich in carbohydrate, whereas 1.6% assumed that high protein should be consumed in a diabetic diet. Food high in carbohydrate and fats was correctly identified by 48% and 42% of the participants, respectively; while sugar-free food was identified by just 18.4% of the sample. Adequate awareness for the blood testing techniques was observed for 66.4% of the individuals, while only 44% were able to explain HbA1c. An adequate level of knowledge was observed among the participants for the effect of exercise (88.3%) or infection (73.1%) on levels of blood glucose. A total of 73.3% of the participants showed adequate levels of awareness for foot care while comorbidity awareness was found to range from 54.9 to 79.8% (Table 3).

The Kruskal-Wallis analysis was performed to understand the correlation between glycemic control as measured by HbA1c levels and demographic variables including age, education level, gender, and employment type. The null hypothesis for the analysis implies that there is no dependence of HbA1c levels on the variable. The null hypothesis was rejected if the critical chi-squared value was less than the *H* statistic. Based on the findings, the null hypothesis was rejected for gender, age, educational level, and employment type (Table 4).

## 4. Discussion

Healthcare practices have always been a matter of interest for various medical professionals. In the present time, a greater number of people are suffering from diabetes from a very young age. Knowledge related to diabetes can help patients to overcome the problem through healthy diet and other important safety measures. This pinpoints the idea that to which extent Saudis are aware of the knowledge related to diabetes. The findings of this study illustrate that the majority of the patients were male suffering from diabetes. The results proposed by this study contradict those provided by Fareed et al. [5]. Accordingly, the majority of the females were affected by poor glycemic controls within the region of Saudi Arabia. The reason behind the problem was the high-level consumption of unhealthy diet. It was further observed that the dietary pattern followed by the diabetic patients was unhealthy due to high-level consumption of fats, cholesterol, sodium, and free sugar.

The majority of the low level of knowledge among diabetes patients was recorded in middle age groups. Besides this, the majority of the patients had a family history of glycemic control, which thus served as one of the major causes of diabetes among patients. Alzaheb and Altemani [21] supported the idea where the majority of the patients belonging to urban areas have poor knowledge about glycemic controls. However, people with substantial background of poor glycemic controls were at greater risks of diabetes. The lack of knowledge regarding diabetes resulted in inefficient healthcare management, as patients with maximum diabetic durations were not habitual of regular exercises along with dietary controls.

Another important finding of this study indicated that the majority of diabetes patients were on medication, while few of them were recommended oral medicines. However, the diabetic population was rarely habitual in consuming a combination of insulin and oral therapy. Nguyen et al. [22] proposed slightly different results that almost half of the diabetic population was using oral medication. The study further indicated that level of HRQoL was low among patients consuming regular insulin rather than healthy dietary therapies. This pinpoints that both insulin and oral medicines can further create an impact on health conditions of diabetes patients. Besides this, the study indicated that the level of knowledge among various Saudi patients were seemingly high. This indicates that the level of awareness among patients served as an important determinant among diabetic patients. Sami et al. [23] indicated that poor quality of knowledge was observed among diabetic patients living in Saudi Arabia, in comparison to those living in any other states. Besides this, low level of knowledge was observed regarding the complications provided by diabetes. However, most of the people were unaware of the importance of medicines to control adverse effects of diabetes.

Other than this, knowledge related to the diabetic diet was not recorded among the majority of Saudi patients, while 50% of the patients were aware of the significance of healthy diet for controlling diabetes. For the majority of patients, a healthy diet is related to the high-level consumption of carbohydrates, whereas for others high protein must be consumed to control the effect and level of diabetes. Alramadan et al. [6] demonstrated that the majority of the people were prone to unhealthy lifestyles leading towards other health-related issues such as obesity. This has provided them greater difficulties in controlling their sugar and blood levels. It was further identified that the majority of patients were not consuming a healthy diet that includes daily consumption of fruits and vegetables, maximizing the risks of inadequate glycemic controls. Similarly, physical activity was also uncommon among diabetes patients. The study argued that in various countries, young people are at greater risks of poor glycemic control.

In addition to this, knowledge related to the blood testing techniques, effects of exercise, and risks associated with diabetes were found in the majority of patients, while only few of them were unable to explain the causes and effects of diabetes. Ansari et al. [24] argued that the important knowledge and safety health measures regarding poor glycemic controls were not accessible to the majority of diabetes patients in Saudi Arabia. Similarly, more than half of the diabetes patients were not following any special diet to refrain from any further health issues. Only a limited number of people were aware of the importance of control diet. This stresses the idea that still the concept related to the knowledge and healthcare measures is unclear among the majority of the patients. The results of this study demonstrated that the level of knowledge among Saudi patients was significant in comparison to results proposed by the aforementioned studies. Still, there is a need to spread valuable knowledge towards patients that are at greater risks of diabetes.

The study has reported an association of several demographic characteristics of patients with their knowledge about HbA1c levels. The higher levels of education among patients followed by the job status of the participants were strong factors for better HbA1c level knowledge, which was also supported by previous studies conducted in low- and middle-income countries [25–27]. Another study has asserted that patients' educational level was a substantial predictor towards their self-care practices [28]. Furthermore, improving knowledge and awareness and pharmacological intervention among diabetic patients and their family members will lead to effective health-related consequences. The findings of this study suggested that the contents of an educational intervention can be developed considering the educational status of the patients as mostly patients with low education level had poor disease knowledge. Secondly, job nature of the diabetic patients must be taken into consideration while informing diabetes education as the disease knowledge of the majority of participants was relatable to that of illiterate participants.

## 5. Conclusion

Saudi Arabia, despite high-level economic growth and development, is prone to health-related risks such as diabetes that serves as the root cause for the development of further diseases. As stated above, currently, people are at major risk of death due to diabetes and its consequences that are now highly developing among diabetes patients. The intake of regular insulin is the major prevention for further effects of diabetes. In various countries, a greater amount of money is invested in monitoring and catering to health-related issues. This indicates the need for better understanding among patients related to their healthcare.

As per the designed questionnaire, it was demonstrated that people suffering from diabetes had adequate knowledge related to the importance of healthy diet to control further effects of the disease. However, it was found that most of the patients are more towards using medicines while a limited number of patients use insulin. Other than this, healthy practices, i.e., exercise, were not common in the majority of the population, while the diet was mostly related to the greater consumption of fats. The glycemic levels were found to be low among the patients; however, limited people were aware of the important measures that are needed to be followed among diabetes patients. The study further indicated that in most of the patients, belonging to families with diabetic background was one of the major causes of its widespread among several other patients. Thus, spreading more knowledge related to diabetes and poor glycemic controls to control the transfer of the disease was recommended. It is recommended that future researchers conduct a study regarding the prevalence of the problem in various urban and rural areas of Saudi Arabia to obtain valuable knowledge.

## Figures and Tables

**Table 1:** Patient demographic characteristics.

Variables	Percentage
Gender	
Male	53.1%
Female	46.9%
Nationality	
Saudi	98.5%
Non-Saudi	1.3%
Data unavailable	0.2%
Age	
23-30 years	1.8%
31-40 years	10.1%
41-50 years	22.1%
51-60 years	39.5%
61-70 years	16.4%
71-80 years	10.1%
Marital status	
Married	93.3%
Single	6.7%
Family history	
Present	71.9%
Absent	28.1%
Education	
Primary level	29.2%
Intermediate level	16.8%
Secondary level	27.1%
College degree	26.9%
Employment type	
Government sector	23.9%
Private sector	3.2%
Self-employment/business	0.4%
Retired	38.9%
Nonworking	33.6%

**Table 2:** Information from patients' records.

Variables	Percentage
Treatment type	
Dietary intervention	5.0%
Oral medication	44.8%
Insulin therapy	13.8%
Both oral and insulin therapy	36.4%
Sought diabetes education	
Yes	71.3%
No	28.7%
Year since diagnosis (median years since diagnosis = 11 years)	
≤5 years	26.8%
6-15 years	46.0%
16–30 years	24.7%
>30 years	2.5%
Hb1AC levels (mean = 9.3 ± 4.6)	
Good control: <7.0%	14.0%
Moderate control: 7.0–8.5%	1.8%
Poor control: >8.5%	53.3%

**Table 3:** Awareness percentage of the participants.

MDKT items	Percentage
N1	51.5
N2	47.8
N3	42.1
N4	18.4
N5	44.6
N6	66.4
N7	42.5
N8	37.7
N9	88.3
N10	73.10
N11	73.30
N12	79.80
N13	65.30
N14	54.90

**Table 4:** Association between levels of HbA1c and sociodemographic variables.

Variables	Value of *H* statistic	Critical chi-squared value	*P* value
Gender	5.47	3.84	<0.001
Nationality	17	9.5	>0.001
Age	3.9	11.07	<0.001
Education level	13.5	7.8	<0.001
Employment type	33	9.48	<0.001

## Data Availability

The data used to support the findings of this study are available from the corresponding author upon request.

## Supplementary Materials

Supplementary Materials The first table is the questionnaire used to collect demographic data. The second table contained the 14-item MDKT questionnaire used to assess patients' knowledge on diabetes with the correct answer marked with an asterisk.
