# Supplementary material for: Knowledge about Diabetes and Glycemic Control among Diabetic Patients in Saudi Arabia
Source: J Diabetes Res. 2020 Mar 7;2020:1239735. doi: 10.1155/2020/1239735 (PMC7081031; doi:10.1155/2020/1239735)
Supplement: Supplementary Materials — The first table is the questionnaire used to collect demographic data. The second table contained the 14-item MDKT questionnaire used to assess patients' knowledge on diabetes with the correct answer marked with an asterisk. [file 1239735.f1.pdf]

APPENDICES

Table A1- Questionnaire used for collection of demographic data of the participants (English).

<table>
<tr><td colspan="7">Choose the answer you think is correct by marking (✓) in the space (only one answer per question)</td></tr>
<tr><td rowspan="2">1</td><td rowspan="2">Gender</td><td>Male</td><td>Female</td><td colspan="3" rowspan="6"></td></tr>
<tr><td>0</td><td>1</td></tr>
<tr><td>2</td><td>Age/years</td><td colspan="2">-------years</td></tr>
<tr><td rowspan="2">3</td><td rowspan="2">Nationality</td><td>Saudi</td><td>Non-Saudi</td></tr>
<tr><td>0</td><td>1</td></tr>
<tr><td rowspan="2">4</td><td rowspan="2">Marital status</td><td>Married</td><td>Not married</td></tr>
<tr><td>0</td><td>1</td></tr>
<tr><td rowspan="2">5</td><td rowspan="2">Job type:</td><td>Governmental</td><td>Non-governmental</td><td>Free business</td><td>Retired</td><td>Not working</td></tr>
<tr><td>0</td><td>1</td><td>2</td><td>3</td><td>4</td></tr>
<tr><td rowspan="2">6</td><td rowspan="2">Educational level:</td><td>Primary</td><td>Intermediate</td><td>Secondary</td><td colspan="2">University</td></tr>
<tr><td>0</td><td>1</td><td>2</td><td colspan="2">3</td></tr>
<tr><td>7</td><td>Duration of diabetes years:</td><td colspan="2">-------years</td><td colspan="3"></td></tr>
<tr><td rowspan="2">8</td><td rowspan="2">Type of treatment:</td><td>Diet only</td><td>Oral</td><td>Insulin only</td><td colspan="2">Oral and insulin</td></tr>
<tr><td>0</td><td>1</td><td>2</td><td colspan="2">3</td></tr>
<tr><td rowspan="2">9</td><td rowspan="2">Diabetes educations:</td><td>Yes</td><td>No</td><td colspan="3" rowspan="3"></td></tr>
<tr><td>0</td><td>1</td></tr>
<tr><td>10</td><td>Family</td><td>Yes</td><td>No</td></tr>
</table>

	**history of diabetes mellites:**	**0**	**1**	

Table A2- Questionnaire used for collection of demographic data of the participants (Arabic).

اختر الإجابة التي تعتقد أنها صحيحة بوضع علامة (✓) في الفراغ (إجابة واحدة فقط لكل سؤال).					
1	الجنس:	ذكر	انثى		
		0	1		
2	العمر/بالسنوات:	---------(سنة)			
3	الجنسية:	سعودي	مقيم		
		0	1		
4	الحالة الاجتماعية:	متزوج	غير متزوج		
		0	1		
5	نوع الوظيفة:	قطاع حكومي	قطاع خاص	اعمال حرة	متقاعد	بدون
		0	1	2	3	4
6	المستوى التعليمي:	ابتدائي او اقل	متوسط	ثانوي	جامعي	
		0	1	2	3	
7	مدة الإصابة بمرض السكري/ بالسنوات:	---------(سنة)				
8	الطريقة التي تستعملها بالمعالجة:	حمية فقط	حبوب	انسولين فقط	حبوب وانسولين	
		0	1	2	3	
9	هل يوجد من أفراد أسرتك مصاب بالسكري؟	نعم	لا			
		0	1			
10	هل سبق تثقيفك عن داء السكري؟	نعم	لا			
		0	1			

Table A3- The 14-item MDKT questionnaire, used for assessing patients' general knowledge on diabetes, with correct answers marked with an asterisk (English).

Michigan Diabetes Research and Training Center's Revised Diabetes Knowledge Test<br>Choose the answer you think is correct by marking (✓) in the space (only one answer per question)					
1	The diabetes diet is:	the way most people eat	a healthy diet for most people	too high in carbohydrate for most people	too high in protein for most people
		0	1*	2	3
2	Which of the following is highest in carbohydrate?	Baked chicken	Swiss cheese	Baked potato	Peanut butter
		0	1	2*	3
3	Which of the following is highest in fat?	Low fat (2%) milk	Orange juice	Corn	Honey
		0*	1	2	3
4	Which of the following is a "free food"?	Any unsweetened food	Any food that has "fat free" on the label	Any food that has "sugar free" on the label	Any food that has less than 20 calories per serving
		0	1	2	3*
5	A1C is a measure of your average blood glucose level for the past:	day	week	6-12 weeks	6 months
		0	1	2*	3
6	Which is the best method for home glucose testing?	Urine testing	Blood testing	Both are equally good	
		0	1*	2	
7	What effect does unsweetened fruit juice have on blood glucose?	Lowers it	Raises it	Has no effect	
		0	1*	2	
8	Which should <u>not</u> be used to treat a low blood glucose?	3 hard candies	1/2 cup orange juice	1 cup diet soft drink	1 cup skim milk
		0	1	2*	3
9	For a person in good control, what effect does exercise have on blood glucose?	Lowers it	Raises it	Has no effect	
		0*	1	2	
10	What effect will an infection most likely have on blood glucose?	Raises it	Lowers it	Has no effect	
		0*	1	2	
11	The best way to take care of your feet is to:	look at and wash them each day	massage them with alcohol each day	soak them for one hour each day	buy shoes a size larger than usual
		0*	1	2	3
12	Eating foods lower in fat decreases your risk for:	nerve disease	kidney disease	heart disease	eye disease
		0	1	2*	3
13	Numbness and tingling may be symptoms of:	kidney disease	nerve disease	eye disease	liver disease
		0	1*	2	3
14	Which of the following is usually <u>not</u> associated with diabetes?	vision problems	kidney problems	nerve problems	lung problems
		0	1	2	3*
